# Development of a new rutin nanoemulsion and its application on prostate carcinoma PC3 cell line

**DOI:** 10.17179/excli2016-668

**Published:** 2017-05-31

**Authors:** Mohammad Ahmad, - Sahabjada, Juber Akhtar, Arshad Hussain, - Badaruddeen, Md Arshad, Anuradha Mishra

**Affiliations:** 1 Herbal Bioactive Research Laboratory, Faculty of Pharmacy, Integral University, Lucknow, India; 2 Molecular Endocrinology Laboratory, Department of Zoology, University of Lucknow, Lucknow, India; 3 College of Pharmacy, King Khalid University, Abha, KSA. Formerly, Faculty of Pharmacy, Integral University, Lucknow, India

**Keywords:** rutin, nanoemulsion, water titration method, cell viability, prostate cancer, reactive oxygen species

## Abstract

Biological effects of rutin bioactive are limited due to its poor oral bioavailability and its degradation in aqueous environments. For the purpose of bioenhancement, different nanoemulsion systems of rutin were developed by aqueous titration method using water as dispersion media. The nanoemulsion systems were characterized for surface morphology, droplet size, polydispersity index, zeta potential, *in vitro* release profile and the formulations were optimized. The anticancer potential of optimized nanoemulsion was evaluated by cells viability (MTT) assay, nuclear condensation, and ROS activity using human prostate cancer (PC3) cell line. On the basis of cell viability data the inhibitory concentration (IC_50_) value for optimized nanoemulsion formulation on PC3 cancer cells was found to be 11.8 μM. Fluorescent microscopic analysis and intracellular ROS generation demonstrated significant ROS induction that might lead to triggering the apoptosis pathway. In conclusion, developed nanoemulsion displayed significant efficacy against prostate carcinoma cells.

## Introduction

Cancer chemoprevention using bioactive compounds has attracted increasing attention in recent years (Liu, 2013[23]; Surh, 2003[38]). Bioactive compounds with anticancer properties alone or in combinations with other potent synthetic molecules are used to prevent or reverse the processes of carcinogenesis, furthermore to minimize the undesirable side effects, which are commonly associated with current therapies (Pan and Ho, 2008[29]). Several bio-flavonoids found to be effective in *in vitro *studies at a particular concentration, often exhibit lower responses at a higher concentration as revealed by *in vivo* studies (Scalbert et al., 2005[31]; Amidon et al., 1995[2]). Many factors such as gastric residence time, permeability, and solubility within the gut and some other factors also direct the bioavailability of bio-flavonoid. Gastrointestinal pH, enzymes, and presence of other nutrients sometimes also influence the stability of these bioactives. Poor aqueous solubility and low dissolution rates of flavonoids contribute to their insufficient bioavailability (Kaur and Kaur, 2014[16]). Rutin was first prepared and identified from the plant *Ruta graveolens* L. (Rutaceae) by August Weiss, a pharmacist-chemist of Nuremberg, Germany in 1842. So its name comes from the name of plant *Ruta graveolens*, a species of Ruta grown as an ornamental plant commonly known as rue or herb of grace (Biçer and Özdemir, 2014[3]; Couch et al., 1946[7]). Rutin (quercetin-3-O-rutinoside) is abundantly found in dietary foods such as grains, fruits (especially in citrus fruits) and berries (Macedo et al., 2014[25]; Ghiasi et al., 2012[11]; Krewson and Couch, 1950[17]). Rutin from buckwheat was isolated by Schunck in 1860 and afterward chemists found rutin in a number of plants. Rutin was first medicinally used for the management of increased capillary fragility, a condition in which the smallest blood vessels become abnormally fragile and rupture, so that small hemorrhages occur. The correction of the capillary fault is known as the "vitamin P" action. According to the scientist James F. Couch, rutin might possess a "vitamin P" action. The term “Vitamin P” had been postulated in 1936 by a Hungarian biochemist, Albert Szent-Györgyi, to account for certain medical effects produced by citrus extracts that could not be explained by reference to ascorbic acid or vitamin C (Grzybowski and Pietrzak, 2013[12]; Couch, 1951[6]; Krewson and Couch, 1950[17]). Various studies conducted both *in vitro *and *in vivo *suggest the diverse pharmacological activities of rutin bioactive including anticancer properties (Naif Abdullah Al-Dhabi et al., 2015[27]; Sharma et al., 2013[34]; Lin et al., 2009[21]). Due to the poor bioavailability, the oral use of rutin is limited and subsequently higher doses are required to achieve the desired anticancer effect. It appears that only about 17 % of an ingested dose is absorbed (Marzouk et al., 2007[26]).

For the purpose of bio-enhancement of drug action, various approaches including nano emulsification, solid dispersions, microemulsions, and development of solid-lipid nanostructures have been undertaken. Nanoemulsification serves as a means to improve the oral bioavailability of lipophilic compounds as well as their ability to improve drug solubility, membrane transport, and absorption *via* the lymphatic system with bypassing first pass metabolism (Gupta et al., 2013[13]). Nanoemulsion offers several advantages over the conventional drug delivery systems including higher solubilization capacity, rapid onset of action, reduced inter-subject differences (Rahman et al., 2011[30]). The nanoemulsions can thus be defined as thermodynamically stable, transparent (or translucent) dispersions of oil and water stabilized through an interfacial film of surfactant molecules having a droplet size less than 100 nm. The development of effective formulation for drugs has long been a major subject, because drug efficacy can be strictly limited by instability and poor solubility in the vehicle. Nanoemulsions have a higher solubilization capacity than conventional micellar solutions. Thermodynamic stability of nanoemulsions offers advantages over unstable dispersions, such as emulsions and suspensions, because they can be manufactured by applying very little energy input (heat or mixing) and have a long shelf life. Nanoemulsions are also reported to make the plasma profiles and bioavailability of many drugs highly reproducible (Lawrence and Rees, 2012[19]; Eccleston, 1994[8]).

Cancer is an increasing health problem worldwide; however, prostate carcinoma is the most common cancer in man and the second leading cause of death in many countries (Kumar et al., 2006[18]; Ullen et al., 2005[39]). Hence, novel therapies are required for the treatment of advanced prostate cancer and to improve patient compliance. Selection of the suitable *in vitro* techniques in cancer research is critical for the evaluation of various genetic, epigenetic and cellular changes. Additionally, the application of the cell line models already exists as a part of the ongoing research on cancer therapy (Louzada et al., 2012[24]). Cell line models are very useful tools for the measurement of mediators such as proliferation deregulation, apoptosis and cancer progression (Vargo-Gogola and Rosen, 2007[40]). To date there are 4 well studied Human Prostate Cancer cell lines. They are DU145, PC3, LNCaP and TSU-PnI. Out of all these PC3 cell line is easily available and shows most similar progression as that of human prostate cancer. The PC3 and DU145 human prostate cancer cell lines are representative of the earlier type-I ADI prostate cancers. PC3, but not DU145 cells retain the coregulators needed for AR tumor suppressor ability of androgen receptor (Litvinov et al., 2006[22]).

In the present study we selected PC3 cell line of human prostate carcinoma to evaluate the antiproliferative potential of the developed rutin nanoemulsion.

## Materials and Methods

### Chemicals and reagents

Propylene glycol monocaprylate (Capryol 90) and caprylocaproyl macrogol-8-glyceride (Labrasol) (Gattefosse, Gennevilliers, France) were gift samples from Colorcon Asia (Mumbai, India), while propylene glycol monocaprylic ester (Sefsol 218) and Kolliphor RH-40 were a gift sample from Nikko Chemicals (Tokyo, Japan) and BASF, Mumbai, respectively. Diethylenemonoglycol ether (Carbitol), Polyethylene Glycol (PEG-200, 400, 600), Isopropyl myristate (IPM), glycerol triacetate (Triacetin), methanol (HPLC-Grade), and Distilled Water were purchased from E-Merck (Mumbai, India). Polyoxyethylene sorbitan monolaurate (Tween-20), polyoxy ethylene sorbitan monostearate (Tween-60), polyoxyethylene sorbitan monooleate (Tween-80), ethanol, isopropyl alcohol were procured from S.D. Fine Chemicals (Mumbai, India). Fluorescent dye 2,7-dichlorodihydrofluorescein diacetate (DCFH-DA) and 4',6'-diamidino-2 phenylindole (DAPI) were purchased from Sigma Aldrich, USA. Eagle's minimal essential medium (MEM), fetal bovine serum (FBS), MTT (3 (4,5-dimethylthiazol-2-yl)-2,5-diphenyltetrazolium bromide) dye and antibiotic solutions were purchased from Himedia, India. Rutin (97 %) bioactives were purchased from Sigma-Aldrich Chemical Company (St. Louis, Missouri). All the reagents used for study were of analytical grade.

### Cell line and culture

Human prostate adenocarcinoma (PC3) cell line was procured from National Centre for Cell Sciences (NCCS), Pune, India. PC3 cells were maintained in MEM medium supplemented with 2.0 mM L-glutamine, 1.5 g/l NaHCO_3_, 0.1 mM non-essential amino acids, 1.0 mM sodium pyruvate and 10 % (v/v) FBS. Cells were incubated at 37 °C and 5 % CO_2_ incubator.

### Selection of excipients on the basis of solubility

To find out appropriate oils, surfactants and co-surfactant as components of nanoemulsion system with high loading capacity is based on the solubility of poorly soluble drug in oils, surfactants, and co-surfactants, screening of component such as oils (middle chain, long chain and synthetic triglycerides) Triacetin, Isopropyl myristate, Capryol-90, Sefsol 218, Olive oil including surfactants Tween-80, Labrasol, Kolliphor-HS15, Kolliphor-RH40 and co-surfactants such as Transcutol-P, Carbitol, Polyethylene glycol-200, 400 and 600. An excess amount of drug was added in the oils and surfactants as well as co-surfactant and kept in isothermal shaker for 72 h at 25 ± 2 °C temperature to reach equilibrium. Finally samples were removed from the shaker and centrifuged at 3000 rpm for 15 min. The supernatant was taken and filtered through a 0.45 µm membrane filter to remove the remaining insoluble drugs. Concentration of rutin was determined by HPLC. All the excipients selected for formulation were under the GRAS (Generally Regarded as Safe) category. Finally excipients were selected as formulation components on the basis of highest solubility of rutin in different excipient (Wang et al., 2009[41]).

### Method of preparation of nanoemulsion

Nanoemulsion systems were developed using phase titration method keeping water as dispersion media. Pseudoternary phase diagrams were constructed to represent the best ratio of oil, surfactant/co-surfactant (S_mix_), and water. Surfactant and co-surfactant was mixed (S_mix_) in different volume ratios (1:0, 1:1, 1:2, 1:3, 2:1, 3:1, etc). These S_mix_ ratios were chosen to reflect increasing concentrations of co-surfactant with respect to surfactant and increasing concentrations of surfactant with respect to co-surfactant for detailed study of the phase diagrams in the nanoemulsion formation. Mixture of oil with S_mix_ was prepared at different ratios (e.g. 10:0, 9:1, 8:2, 7:3, 6:4, 5:5, 4:6, 3:7, 2:8, 1:9, 0:10) into different vials. A small amount of water in 5 % (w/w) increments was added into the vials. Following each water addition the mixture in vials was centrifuged for 2 to 3 minute and incubated at 25 °C for 48 h with gentle shaking. The resulting mixtures were evaluated by visual and microscopy observation. For phase diagram the nanoemulsion is the region of clear and isotropic solution. The physical state of the nanoemulsion is marked on the phase diagrams with one axis representing aqueous phase, the second representing oil and the third representing a mixture of surfactant and co-surfactant (S_mix_) at fixed weight ratios (S_mix_ ratio). The nanoemulsion area in each phase diagram is plotted and the wider region indicated the better self nanoemulsifying efficiency (Singh et al., 2008[37]).

### Construction of pseudoternary phase diagrams

**Figure 1:**
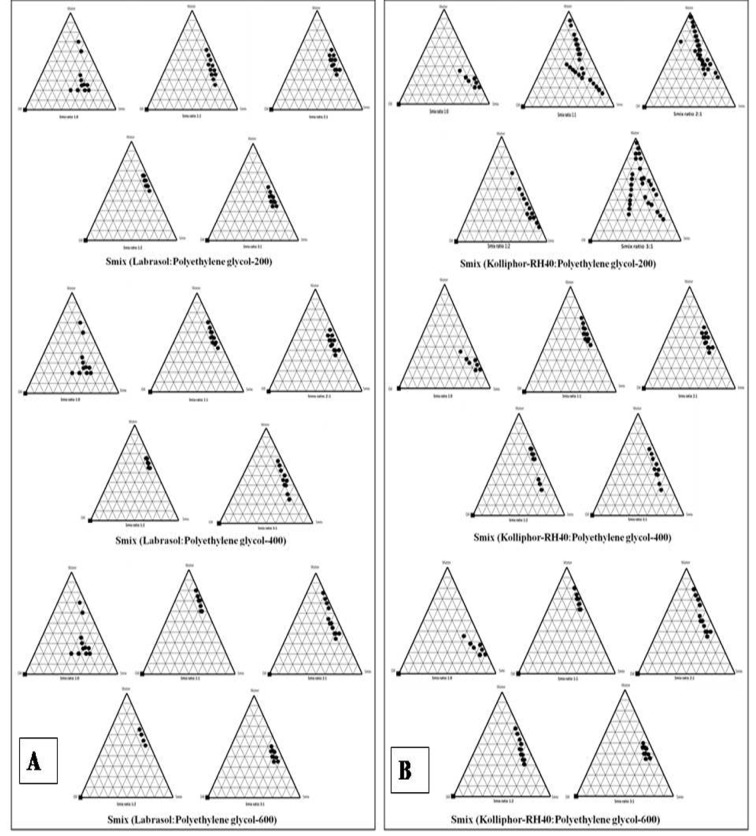
Ternary phase chart indicating o/w nanoemulsion region at different S_mix_ ratios. (A) Isopropyl myristate, S_mix _(Labrasol and polyethylene glycol) and water, (B) Sefsol 218, S_mix_ (Kolliphor-RH40 and Polyethylene glycol) and water

Pseudoternary phase diagrams were drawn for determining S_mix_ ratio and its ratio with oil phase in emulsion system which gives the nanoemulsion region (Figure 1(Fig. 1)). On the basis of solubility and compatibility of rutin with various excipients, Kolliphor RH-40/Labrasol//PEG-200, 400, 600 was selected as the surfactant, PEG-200, 400, 600 as co-surfactant and Sefsol218/Isopropyl myrsitate were selected as the oil phase. For every phase diagram, oil and accurate S_mix_ ratio was mixed in volume ratios ranging from 1:9 to 9:1 to obtain sixteen different combinations like 1:9, 1:8, 1:7, 1:6, 1:5 1:4, 1:3.5, 1:3, 3:7, 1:2, 4:6, 5:5, 6:4, 7:3, 8:2 and 9:1 and titrated with water for constructing zone of nanoemulsion. S_mix_ ratios 1:0, 1:1, 2:1, 1:2 and 3:1 were selected from above experiments. Concentrations of all the excipients used were within permissible limits recommended for oral usage.

### Development of drug containing formulations

Nanoemulsion system was developed by taking best suited optimized concentrations of oil phase (Sefsol 218), surfactant (Kolliphor RH-40), and co-surfactant (PEG-400) was selected from ternary phase diagrams and this system was then loaded with excess amount of rutin (10 mg/ml). Another system comprising the oil phase (Isopropyl myristate), surfactant (Labrasol) and co-surfactant (PEG-600) was selected from ternary phase diagrams and this system was then loaded with excess amount of rutin (10 mg/ml). These were subsequently sonicated for 10 minutes on a bath sonicator.

### Measurement of drug contents in nanoemulsion

Finally six formulations were selected and the best suitable formulations amongst them were analyzed for the drug contents from nanoemulsion formulations that were extracted in methanol. The solutions were filtered, using Whatman filter paper of 0.45 µm pore size and analyzed for the drug contents through HPLC.

### Characterization of optimized formulation dispersibility test

One ml of each nanoemulsion formulation was added to 500 mL of distilled water and 0.1 N HCl separately at a temperature of 37 °C in a standard USP XXII dissolution apparatus. The dissolution paddle rotated at a speed of 50 rpm to provide gentle mixing. *In vitro *performance of the formulations was visually assessed using grading system as per the dispersibility comments. Formulations that approved the thermodynamic stability test and dispersibility test in Grade A as well as B had been chosen for additional studies (Akhtar et al., 2014[1]).

### Thermodynamic stability studies

Selected formulations were subjected to different thermodynamic stability tests to assess their physical stability. For centrifugation test, a definite volume of formulations were diluted in (1:10-1:100 ratios) with aqueous medium and centrifuged at 15,000 rpm for 15 minutes, then the formulation was observed visually for the phase separation. After that formulations were subjected to freeze thaw cycles between −21 °C and +25 °C, with formulation storage at each temperature for not less than 48 h. Those formulations found to be thermodynamically stable were selected for further characterization (Shafiq-un-Nabi et al., 2007[33]).

### Percentage transmittance

Percentage transmittance of the prepared nanoemulsion was determined spectrophotometrically after 100 times dilution with methanol using Shimadzu UV-visible spectrophotometer keeping distilled water as a blank.

### Droplet size and zeta potential analysis

The droplet size and the size distribution of the prepared nanoemulsion systems were determined using Zetasizer (Nano ZS, Malvern Instruments, U.K). For the purpose of particle size different dilution were made and each sample was analyzed in triplicate at a temperature, 20 °C and refractive index, 1.4. Zeta potential was also measured by photon correlation spectroscopy. The prepared nanoemulsions with and without dilution were analyzed using double distilled water. 

### Transmission electron microscopy (TEM)

Details on the morphology and other structural features of the nanoemulsion formulations were viewed using Transmission Electron Microscope (TEM), Model EM-410 LS facility available at USIF, AMU, Aligarh (India). Prior to the analysis, the samples were diluted at 10-100 times with water and applied on the grids which were stained with 2 % (w/v) phosphotungstic acid for 30 s and then grids were observed after drying, using combination of bright field imaging at increasing magnification to reveal the form and size of nanoemulsion droplets (Shafiq-un-Nabi et al., 2007[33]).

### Viscosity

Viscosity of the selected formulations were determined at 25 ± 0.5 °C by Brookfield Viscometer DV III ultra V6.0 RV cone and plate rheometer (Brookfield Engineering Laboratories, Middleboro, MA) (Shafiq-un-Nabi et al., 2007[33]).

### In vitro drug release study

Comparative *in vitro* release profile of the optimized nanoemulsion and the pure drug suspension in phosphate buffer (pH 6.8), 0.1 N HCl (pH 1.2) and distilled water were studied using dialysis bag techniques at 100 rpm rotational speed. The temperature was maintained at 37 ± 0.5 °C. Drug release was carried out by placing 1 ml of nanoemulsion in treated dialysis bag (MWCO 14,000 g/mole, Sigma, USA). 0.5 mL aliquots was withdrawn at pre-determined time intervals (0, 0.25, 0.5, 0.75, 1, 1.25, 1.5, 1.75, 2, 2.25, 2.5, 2.75, 3, 4, 6, 8, 12, 16, 20, and 24 h) and same volume was replenished with fresh dissolution media to maintain the sink condition. The samples withdrawn were filtered using 0.45 µm filter paper and the drug was analyzed by reported HPLC method (Shafiq-un-Nabi et al., 2007[33]). 

### In vitro cell viability assay

To detect cell viability after treatment with rutin nanoemulsion, approximately 1x10^4^ cells/well of PC3 were seeded in 100 μl complete culture medium in 96-well culture plate and incubated overnight in humidified air. Stock was prepared in phosphate buffer saline (PBS) and diluted into culture medium to the desired concentrations 2, 5, 10, 15 and 20 μM, then added to the wells. After 24 h of incubation period, 10 μl of MTT (5 mg/ml in PBS) reagent was added and re-incubated at 37 °C until purple formazan crystals developed. Formazan blue crystals were dissolved in 100 μl of DMSO and read at 540 nm using microplate ELISA reader (BIORAD 680, USA). The plot of percent cell viability versus nanoemulsion concentrations was used to calculate the concentration lethal to 50 % of the cells (IC_50_). The cellular morphological changes were observed under inverted phase contrast microscopy (Nikon ECLIPSE Ti-S, Japan) (Siddiqui and Arshad, 2014[36]).

### Intracellular ROS activity

Intracellular ROS generation was analyzed by using fluorescence microscopic imaging technique (Siddiqui et al., 2015[35]). Cells (1×10^4 ^per well) were exposed at two effective concentrations i.e. 5 and 10 μM of rutin nanoemulsion for 12 h. Subsequently, cells were incubated with DCFH-DA (10 mM) at 37 °C for 30 min and washed with PBS. Intracellular fluorescence intensity of cells was visualized by inverted fluorescent microscope (Nikon ECLIPSE Ti-S, Japan). For quantitative fluorometric analysis, cells (1×10^4^ per well) were seeded and treated with nanoemulsion in 96-well black bottom culture plate. Fluorescence intensity was measured with a multiwell microplate reader (Synergy H1 Hybrid Multi-Mode Microplate Reader, BioTek, USA) at an excitation wavelength of 485 nm and at an emission wavelength of 528 nm. Data were expressed as percentage of fluorescence intensity relative to the control wells.

### Apoptotic effect of formulation using DAPI stain

Fluorescent nuclear dye was used to analyze the apoptotic effect of rutin nanoemulsion. PC3 cells (1×10^5^ cells per well) were seeded in 24-well culture plate overnight and treated with rutin nanoemulsion for 24 h. Following incubation period, cells were washed and fixed in 4 % paraformaldehyde for 15 min followed by permeabilization with permeabilizing buffer (3 % paraformaldehyde and 0.5 % Triton X-100) for 10 min. After staining with DAPI dye (50 µg/ml), images of condensed nuclei undergoing apoptosis were captured with an inverted fluorescent microscope (Nikon ECLIPSE Ti-S, Japan). Apoptosis was quantitated by morphological changes of nuclei with approximately 500 cells/well representing one sample (Siddiqui et al., 2015[35]).

### Statistical analysis 

The results were expressed as mean ± SD and were analyzed statistically (graph pad prism for Windows, version 5) using one-way analysis of variance (ANOVA) followed by Tukey's test and considered statistically significant when *p* < 0.05.

## Results and Discussion

### Preparation and characterization of nanoemulsion 

**Figure 2:**
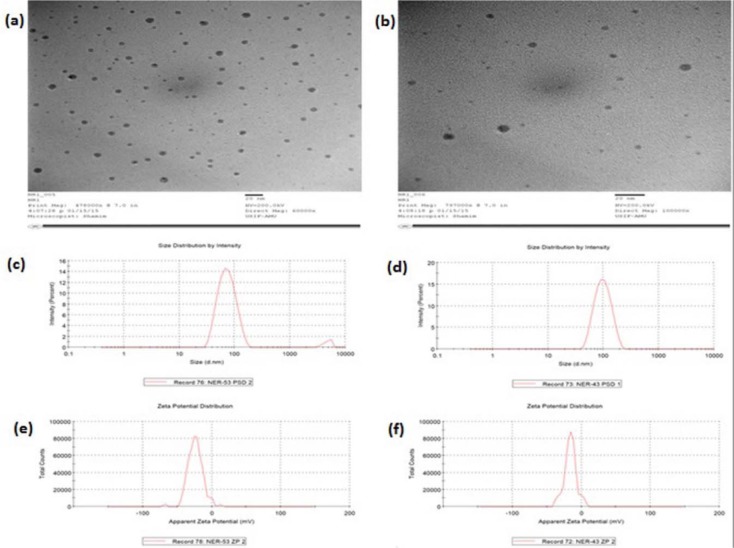
Surface morphology of rutin nanoemulsion systems by TEM. (a) RF-13, (b) NRF-07; Droplet size distribution, (c) RF-13, (d) NRF-07; Zeta-potential, (e) RF-13 and (f) NRF-07

Pseudoternary phase diagrams were constructed to represent the concentration of oils, surfactant and co-surfactant used for the development of appropriate formulation (Shafiq-un-Nabi et al., 2007[33]). Solubility of Rutin in Isopropyl myristate, Sefsol 218 was highest among all the oils screened (Table 1(Tab. 1)). On the basis of solubility study Labrasol and Kolliphor-RH40 were selected as surfactant whereas polyethelene glycol 600 and polyethelene glycol-400 as co-surfactant respectively. Rutin nanoemulsion system (RF-13) contains Isopropyl myristate (IPM) as oil phase with S_mix_ comprised of Labrasol and PEG-600 whereas another rutin nanoemulsion system (NRF-07) has Sefsol 218 as the oil phase with S_mix_ comprising Kolliphor-RH40 as surfactant and PEG-400 as co-surfactant. Nanoemulsion systems of these optimized components were evaluated for the dispersibility, thermodynamic stability, percentage transmittance and droplet size analysis (Table 2(Tab. 2)). Thermodynamically stable systems are formed at a particular concentration of oil, surfactant and water, without any kinetic instability and phase separation (Table 3(Tab. 3) and Table 4(Tab. 4)). In order to develop an oral nanoemulsion formulation, dispersibility studies were of great importance. Dispersibility tests were done to evaluate the dispersion efficiency and the stability of nanoemulsion systems in the gastrointestinal fluids. On the basis of dispersibility assessment, those formulations in grade A as well as in grade B had been selected for additional studies. The percentage transmittance that provides an idea about the size of the droplets and the droplet size is proportional to the percentage transmittance of the formulation. Percentage transmittance approaching 100 % indicates the isotropicity of formulations. The percentage transmittance for RF-13 formulation was 97.34 % while for NRF-07 formulation was found to be 96.12 % (Table 5(Tab. 5)). Upon optimization, it was observed that formulation RF-13 and NRF-07 have mean droplet size of 70.09 nm, 86.84 and minimum PDI 0.195, 0.165 respectively. The zeta potential of both selected formulation (RF-13 and NRF-07) was between -15mV to -17 mV, which indicated the stability of both the formulation and negatively charged surface of drug carrier. The viscosity of the selected formulations was in the range of 8-20 cps as expected for o/w nanoemulsion. Furthermore, transmission electron microscopy (TEM) was employed to evaluate the morphology and structure of the resulting nanoemulsion droplets. TEM study reveals that most of the nanoemulsion droplets containing rutin were in spherical shape (Figure 2(Fig. 2)).

### In vitro drug release study 

**Figure 3:**
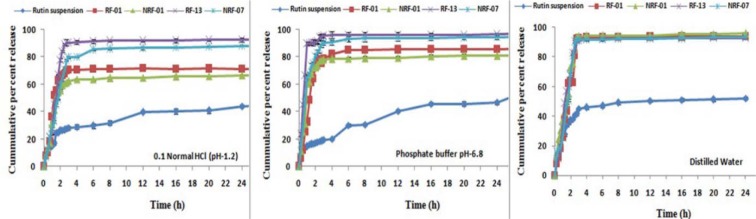
Comparative *in vitro* release profile of prepared nanoemulsion and rutin suspension in Phosphate buffer (pH 6.8); 0.1 N HCl (pH 1.2) and Distilled Water

Dialysis bag technique was used to study and compare the *in vitro *release profile of rutin nanoemulsion and the plain drug. The drug release in 0.1 N HCl (pH 1.2), phosphate buffer (pH 6.8) and distilled water is shown in Figure 3(Fig. 3). The results showed ~ 90 % rutin release from nanoemulsion formulation while pure drug suspension showed ~ 20 % in first 2 h. The release of drug from nanoemulsion formulation was significantly higher (p<0.05) compared to pure drug suspension. 

### Effect of rutin nanoemulsion on percent cells viability and cell morphology

**Figure 4:**
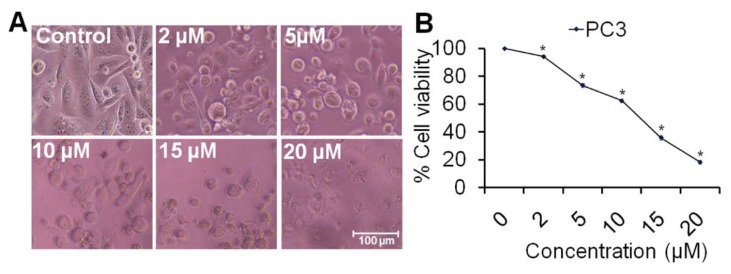
Effect of rutin nanoemulsion on percent cells viability and cell morphology of human prostate carcinoma PC3 cell line. (A) Morphological view of live & dead cells treated with 2, 5, 10, 15 and 25 µM concentration of rutin nanoemulsion. Scale bar=100 μm. (B) Percent cell viability of PC3 cells measured by a MTT assay at 24 h as mentioned in the experimental part. Values are expressed as mean ± SEM of at least three independent experiments, *p < 0.05 as compared with their respective control

The nanoemulsion systems were tested for *in vitro* antiproliferative activity against PC3 (prostate cancer) cells using MTT reduction assay. Cellular morphological changes were observed significantly in PC3 cells 24 h after the treatment of compound at different concentration by inverted phase contrast microscopy. Figure 4A(Fig. 4) showed that the cells drastically changed their morphological shapes to round shape in treatment groups depending upon dose which is characterized by condensed cytoplasm, cellular shrinkage and clumped cells forming clusters (Kumar et al., 2006[18]). The cell viability data as cleared from Figure 4B(Fig. 4) indicates that 2 μM of nanoemulsion reduces the cell viability approximately 90.14 % (*p*<0.05) as compared with control and it was dramatically reduced to approximately 73.31 % (*p *< 0.05) when treated with 5 μM of rutin nanoemulsion. However, 10 μM of compound reduces the viability approximately 62.3 % and it was noticeably reduced to approximately 35.61 % (*p *< 0.05) at 15 μM of rutin nanoemulsion. Moreover, 20 μM of rutin nanoemulsion reduces PC3 cells viability only 18.15 %. A similar study has shown the toxicity of the oligonucleotide/cationic nanoemulsion complexes on liver HepG2 cells reducing cell numbers significantly (Fraga et al., 2008[10]). Present results suggested that treatment of rutin nanoemulsion significantly reduces the cell viability of cancer cell line in a dose-dependent manner.

### Rutin nanoemulsion induces intracellular ROS generation

**Figure 5:**
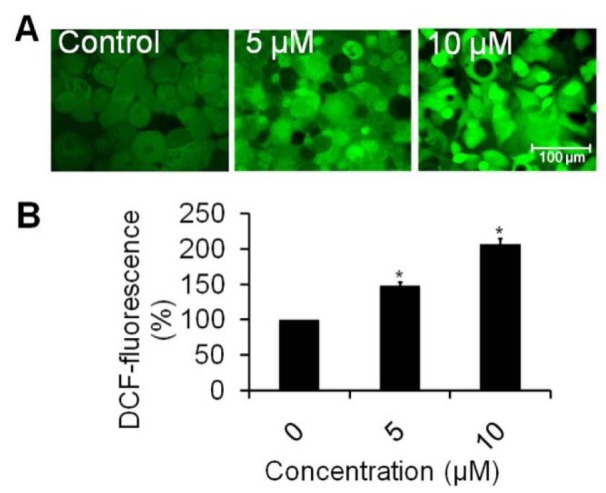
Effect of rutin nanoemulsion on intracellular ROS level in comparison to untreated cells against human prostate carcinoma PC3 cell line. (A) Photomicrographs showing intracellular ROS generation in PC3 cells induced by 5 and 10 µM concentration of rutin nanoemulsion after 12 h incubation and stained with DCFH-DA. Scale bar=100 μm. (B) Graph showing extent of ROS generation expressed as the percentage of fluorescence intensity relative to the control. Values are expressed as mean ± SEM of at least three independent experiments, *p < 0.05 as compared with their respective control

As observed from Figure 5A(Fig. 5), PC3 cells treated with rutin nanoemulsion showed considerable increase in ROS intensity in a dose-dependent manner as compared to untreated cells. The results of quantitative measurement of ROS level showed that 5 µM of rutin nanoemulsion induced 148.56 % (*p *< 0.05) enhancement in ROS production as compared to control. Moreover, ROS production was increased by 207.57 % (*p *< 0.05) at 10 µM of rutin nanoemulsion when compared to untreated cells (Figure 5B(Fig. 5)). During apoptosis, ROS are produced by mitochondria which increase the mitochondrial membrane permeability and lead to the apoptotic phenotype (Fleury et al., 2002[9]). ROS are more reactive than molecular oxygen which may be associated with the activation of signal molecules and destabilization of mitochondrial membrane inducing the release of apoptotic agents resulting in toxicity to cancer cells (Chibber et al., 2012[5]). Our results clearly stated that rutin nanoemulsion provoked cells' death by inducing ROS production.

### Rutin nanoemulsion induces apoptosis 

**Figure 6:**
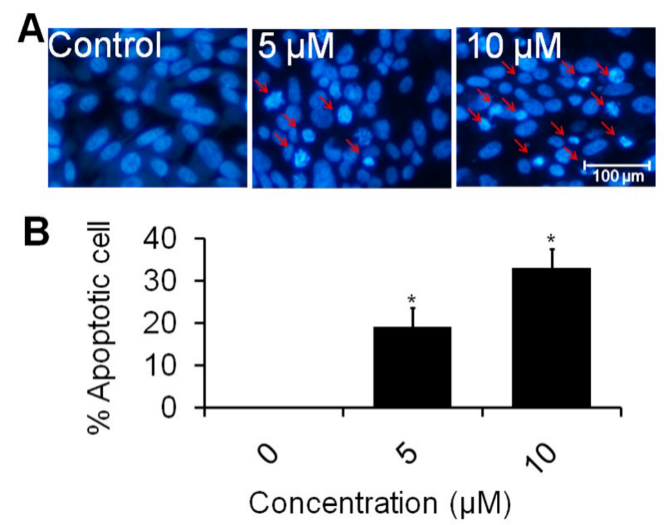
Quantitative measurement of apoptotic cells in human prostate carcinoma PC3 cell line. (A) PC3 cells were treated with 5 and 10 µM concentration of rutin nanoemulsion and stained with DAPI. Scale bar=100 μm. (B) Representative graphs showing the numerical data of the percent apoptotic cells against the concentration of rutin nanoemulsion. Values are expressed as mean ± SEM of at least three independent experiments, *p < 0.05 as compared with their respective control.

The treatment by rutin nanoemulsion caused an increased number of apoptotic cells over the control cells. Morphology of treated cells suggested that the mode of action of nanoemulsion against tumor cells was apoptosis. The morphological properties such as cell shrinkage, nuclear fragmentation and condensation of chromatin forming apoptotic bodies were clearly proved (Ouyang et al., 2012[28]). The results of quantitative measurement of apoptotic cells showed that 5 µM of rutin nanoemulsion induced 19 % (*p *< 0.05) apoptotic cells as compared to control. Moreover, percent apoptotic cells were increased by 33 % (*p *< 0.05) at 10 µM of compound when compared to untreated cells (Figure 6B(Fig. 6)). A previous study has shown that a nanoemulsion system incorporating both lycopene and gold nanoparticles significantly induced early apoptosis in HT-29 colon cancer cells (Huang et al., 2015[14]). Similarly, rutin flavonoid has shown cytotoxicity against leukemia, multiple myeloma and melanoma cell lines *in vitro *(Ikeda et al., 2015[15])*.* A previous study has shown that rutin inhibited human leukemia tumor growth in a murine xenograft model system (Lin et al., 2012[20]). Further, *in vitro* study also revealed the apoptotic effect of rutin in neuroblastoma LAN-5 cell line (Chen et al., 2013[4]). In conclusion, the optimized nanoemulsion of rutin showed good thermodynamic stability with significant improvement in the drug release profile as compared to rutin suspension. The nanoemulsion system of rutin bioactive was found to be more efficacious against prostate cancer cell line at very low concentration when compared to pure rutin. Moreover, bio-distribution studies as well as *in vivo* anti-cancer efficacy studies on animal models of cancer are desired to explore the anticancer potential of rutin nanoemulsion.

## Conflict of interest

The authors declare that they have no conflict of interest.

## Acknowledgement

This work was supported by the Uttar Pradesh Council for Science and Technology (File No. CST/YSS/D-1203), Uttar Pradesh, India. We are thankful to Dr. Amit Mishra (Head Pharmaceutics Division) for providing the Malvern-Zetasizer facilities available at Central Drug Research Institute (CSIR-CDRI), Lucknow (U.P.), India. Thanks to Ms. Vaishali Meghare (Indian Commercial Company Pvt. Ltd. Mumbai) for providing Sefsol 218 as a gift samples from Nikko Chemicals Tokyo, Japan. The authors offer their sincere thanks to Dean Research & Development, Integral University for providing technical support and assigning Communication no. (IU/R&D/2017-MCN00082).

## Figures and Tables

**Table 1:**
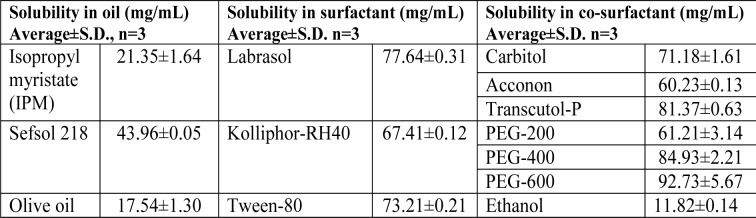
Rutin solubility in different oils, surfactants and co-surfactants

**Table 2:**
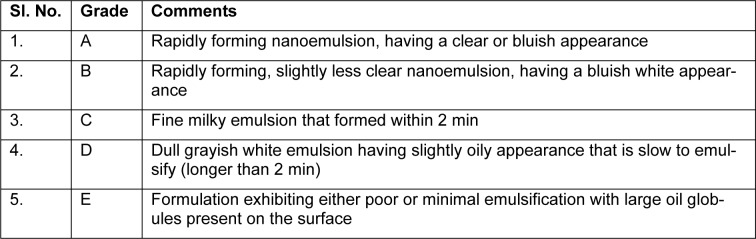
Observation for dispersibility test

**Table 3:**
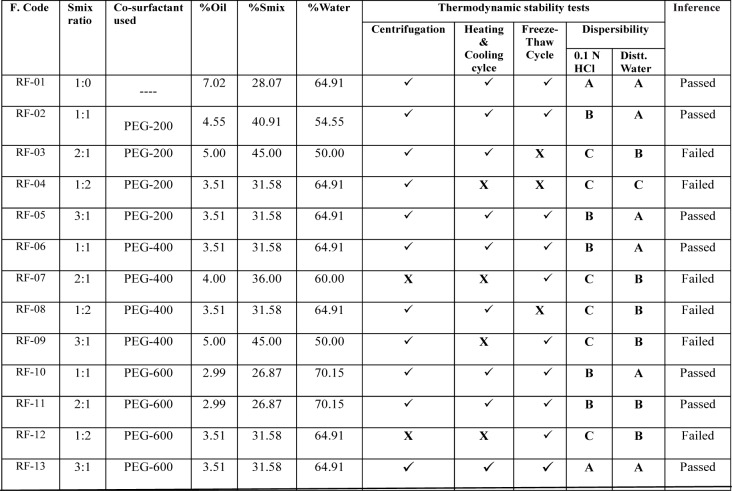
Thermodynamic stability studies of rutin-loaded nanoemulsion containing Isopropyl myristate (Oil phase), Labrasol (Surfactant) and Polyethylene glycol (Co-surfactant)

**Table 4:**
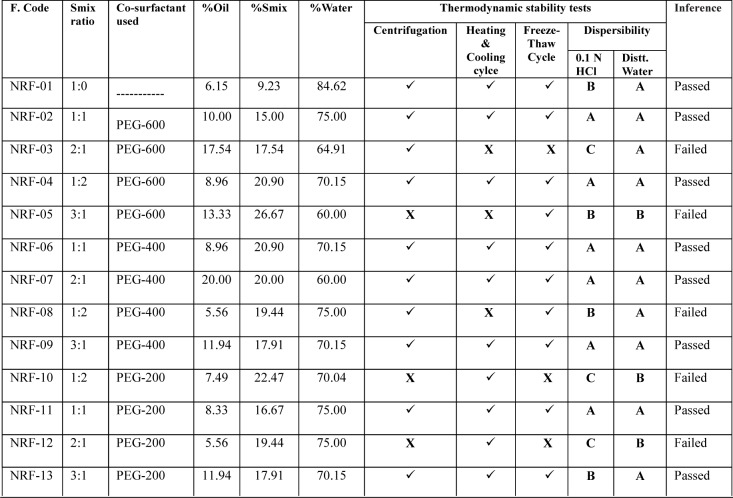
Table 4. Thermodynamic stability studies of rutin-loaded nanoemulsion containing Sefsol 218 (Oil phase), Kolliphor-RH40 (Surfactant) and Polyethylene glycol (Co-surfactant)

**Table 5:**
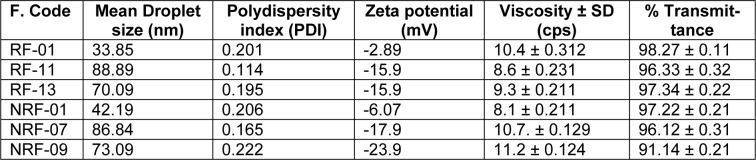
Characterization of optimized rutin nanoemulsion systems

## References

[1] Akhtar J, Hussain Siddiqui H, Fareed S, Aqil M (2014). Nanomulsion as a carrier for efficient delivery of metformin. Curr Drug Deliv.

[2] Amidon GL, Lennernäs H, Shah VP, Crison JR (1995). A theoretical basis for a biopharmaceutic drug classification: the correlation of in vitro drug product dissolution and in vivo bioavailability. Pharm Res.

[3] Biçer E, Özdemir S (2014). Interaction thermodynamics of rutin (Vitamin P) with basic L-amino acids at physiological pH: the effect of temperature on the affinity order. Russ J Electrochem.

[4] Chen H, Miao Q, Geng M, Liu J, Hu Y, Tian L (2013). Anti-tumor effect of rutin on human neuroblastoma cell lines through inducing G2/M cell cycle arrest and promoting apoptosis. Sci World J.

[5] Chibber S, Farhan M, Hassan I, Naseem I (2012). Novel aspect of chemophototherapy in treatment of cancer. Tumor Biol.

[6] Couch JF, United States Department of Agriculture (1951). Rutin a new drug from buckwheat. Crops in peace and war: The yearbook of agriculture, 1950-1951.

[7] Couch JF, Naghski J, Krewson CF (1946). Buckwheat as a Source of Rutin. Science.

[8] Eccleston J, Swarbrick J, Boylan JC (1994). Microemulsions. Encyclopedia of pharmaceutical technology.

[9] Fleury C, Mignotte B, Vayssière JL (2002). Mitochondrial reactive oxygen species in cell death signaling. Biochimie.

[10] Fraga M, Laux M, Rejane dos Santos G, Zandona B, Dos Santos Giuberti C, de Oliveira MC (2008). Evaluation of the toxicity of oligonucleotide/cationic nanoemulsion complexes on Hep G2 cells through MTT assay. Pharmazie.

[11] Ghiasi M, Azadnia A, Arabieh M, Zahedi M (2012). Protective effect of rutin (vitamin p) against heme oxidation: a quantum mechanical approach. Comp Theor Chem.

[12] Grzybowski A, Pietrzak K (2013). Albert Szent-Györgyi (1893-1986): the scientist who discovered vitamin C. Clin Dermatol.

[13] Gupta S, Kesarla R, Omri A (2013). Formulation strategies to improve the bioavailability of poorly absorbed drugs with special emphasis on self-emulsifying systems. ISRN Pharm.

[14] Huang RF, Wei YJ, Inbaraj BS, Chen BH (2015). Inhibition of colon cancer cell growth by nanoemulsion carrying gold nanoparticles and lycopene. Int J Nanomed.

[15] Ikeda NE, Novak EM, Maria DA, Velosa AS, Pereira RM (2015). Synthesis, characterization and biological evaluation of Rutin–zinc (II) flavonoid-metal complex. Chem Biol Interact.

[16] Kaur H, Kaur G (2014). A critical appraisal of solubility enhancement techniques of polyphenols. J Pharm.

[17] Krewson CF, Couch JF (1950). Production of rutin from buckwheat. J Pharm Sci.

[18] Kumar S, Suresh PK, Vijayababu MR, Arunkumar A, Arunakaran J (2006). Anticancer effects of ethanolic neem leaf extract on prostate cancer cell line (PC-3). J Ethnopharmacol.

[19] Lawrence MJ, Rees GD (2012). Microemulsion-based media as novel drug delivery systems. Drug Deliv Rev.

[20] Lin JP, Yang JS, Lin JJ, Lai KC, Lu HF, Ma CY (2012). Rutin inhibits human leukemia tumor growth in a murine xenograft model in vivo. Environ Toxicol.

[21] Lin JP, Yang JS, Lu CC, Chiang JH, Wu CL, Lin JJ (2009). Rutin inhibits the proliferation of murine leukemia WEHI-3 cells in vivo and promotes immune response in vivo. Leuk Res.

[22] Litvinov IV, Antony L, Dalrymple SL, Becker R, Cheng L, Isaacs JT (2006). PC3, but not DU145, human prostate cancer cells retain the coregulators required for tumor suppressor ability of androgen receptor. Prostate.

[23] Liu RH (2013). Dietary bioactive compounds and their health implications. J Food Sci.

[24] Louzada S, Adega F, Chaves R (2012). Defining the sister rat mammary tumor cell lines HH-16 cl.2/1 and HH-16.cl.4 as an in vitro cell model for Erbb2. PloS one.

[25] Macedo AS, Quelhas S, Silva AM, Souto EB (2014). Nanoemulsions for delivery of flavonoids: formulation and in vitro release of rutin as model drug. Pharm Dev Technol.

[26] Marzouk MS, Soliman FM, Shehata IA, Rabee M, Fawzy GA (2007). Flavonoids and biological activities of Jussiaea repens. Nat Prod Res.

[27] Naif Abdullah Al-Dhabi NA, Arasu MV, Park CH, Park SU (2015). An up-to-date review of rutin and its biological and pharmacological activities. EXCLI J.

[28] Ouyang L, Shi Z, Zhao S, Wang FT, Zhou TT, Liu B (2012). Programmed cell death pathways in cancer: a review of apoptosis, autophagy and programmed necrosis. Cell Prolif.

[29] Pan MH, Ho CT (2008). Chemopreventive effects of natural dietary compounds on cancer development. Chem Soc Rev.

[30] Rahman A, Harwansh R, Mirza A, Hussain S, Hussain A (2011). Oral lipid based drug delivery system: formulation, characterization and application: a review. Curr Drug Deliv.

[31] Scalbert A, Manach C, Morand C, Rémésy C, Jiménez L (2005). Dietary polyphenols and the prevention of diseases. Crit Rev Food Sci Nutr.

[32] Schunck E (1860). On the yellow colouring matters obtained from the leaves of Polygonum fagoeyrum, or common buckwheat. Mem Proc Manchester Lit Phil Soc Series 2.

[33] Shafiq-un-Nabi S, Shakeel F, Talegaonkar S, Ali J, Baboota S, Ahuja A (2007). Formulation development and optimization using nanoemulsion technique: a technical note. AAPS Pharm Sci Tech.

[34] Sharma S, Ali A, Ali J, Sahni JK, Baboota S (2013). Rutin: therapeutic potential and recent advances in drug delivery. Expert Opin Investig Drugs.

[35] Siddiqui S, Ahmad E, Gupta M, Rawat V, Shivnath N, Banerjee M (2015). Cissus quadrangularis Linn exerts dose‐dependent biphasic effects: osteogenic and anti‐proliferative, through modulating ROS, cell cycle and Runx2 gene expression in primary rat osteoblasts. Cell Prolif.

[36] Siddiqui S, Arshad M (2014). Osteogenic potential of punica granatum through matrix mineralization, cell cycle progression and runx2 gene expression in primary rat osteoblasts. Daru.

[37] Singh AK, Chaurasiya A, Singh M, Upadhyay SC, Mukherjee R, Khar RK (2008). Exemestane loaded self-microemulsifying drug delivery system (SMEDDS): development and optimization. AAPS Pharm Sci Tech.

[38] Surh YJ (2003). Cancer chemoprevention with dietary phytochemicals. Nat Rev Cancer.

[39] Ullén A, Lennartsson L, Harmenberg U, Lennernäs B, Majumder K, Holmberg AR (2005). Prostate cancer cell lines lack amplification: overexpression of HER2. Acta Oncol.

[40] Vargo-Gogola T, Rosen JM (2007). Modelling breast cancer: one size does not fit all. Nat Rev Cancer.

[41] Wang L, Dong J, Chen J, Eastoe J, Li X (2009). Design and optimization of a new self-nanoemulsifying drug delivery system. J Colloid Interface Sci.

